# Whole egg powder makes nutritious diet more affordable for Ethiopia: A cost of the diet and affordability analysis

**DOI:** 10.1111/mcn.13274

**Published:** 2021-09-23

**Authors:** Kaleab Baye, Andinet Abera, Stanley Chitekwe, Paulos Getachew, Abebe Hailemariam, Filippo Dibari, Arnaud Laillou

**Affiliations:** ^1^ Center for Food Science and Nutrition, College of Natural and Computational Sciences Addis Ababa University Addis Ababa Ethiopia; ^2^ Research Center for Inclusive Development in Africa (RIDA) Addis Ababa Ethiopia; ^3^ Food Science and Nutrition Research Directorate Ethiopian Public Health Institute Addis Ababa Ethiopia; ^4^ Nutrition Section UNICEF Ethiopia Addis Ababa Ethiopia; ^5^ Nutrition Team World Food Program Addis Ababa Ethiopia

**Keywords:** animal source foods, cost‐of‐the diet, diet quality, food price, nonaffordability, nutrient adequacy

## Abstract

Despite sustained nutrition education, consumption of animal source foods (ASFs) has been hindered by their low availability, accessibility and affordability. Drying eggs into powder can reduce transport/storage costs, increase shelf‐life and allow easier dosage for use of smaller portions. This study aimed to evaluate the contribution of integrating egg powder to the nutrient adequacy and affordability of diets. Using the ‘cost of the diet’ analysis, we simulated the incorporation of egg powder into households' and children's diet and evaluated its contribution to the nutrient adequacy and affordability of diets. Analysis of the household consumption and expenditure survey (HCES 2016) revealed that only 0.2% of the total consumption expenditure was allocated for eggs, far below the 2.2% and 4.3% required to allow the consumption of one egg a day by the average and the poorest households, respectively. However, the minimum‐cost nutritious diet required only 2.5 g of egg powder/person/day to reduce the cost of the optimized diet by 14% (0–24%), allowing an additional 1.2 million households (~4–6 million individuals) afford the optimized diet. The optimized diet for a child 6–23 months of age could be afforded by all households, except by those in the poorest wealth quintile. But, free distribution of egg powder to households in the poorest wealth quintile, if supplemented by effective nutrition education, can allow them to afford the minimum‐cost nutritious diet for their 6‐ to 23‐month child. The simple dehydration of egg into egg powder can have a substantial contribution towards increased egg consumption by increasing the affordability of the minimum‐cost nutritious diet.

**Key messages**

- The minimum‐cost nutritious diet is unaffordable for 3/4 of Ethiopian households.
- A 2.5 g of egg powder/person/day reduces the cost of the optimized diet by 14%.
- Egg powders allow an additional ~1.2 million households afford the optimized diet.
- Free provision of egg powder for the poor makes the optimized child diet affordable‐for‐all.

## 1 INTRODUCTION

Malnutrition, in all its forms, is among the leading cause of morbidity and mortality in low‐and‐middle income countries (LMICs). Poor quality diet, partly characterized by low diversity, has been consistently associated with child stunting and micronutrient deficiencies (Krasevec et al., 2017; Moursi et al., 2008). In particular, the consumption of animal source foods (ASFs) in the first 1000 days of life, starting from conception to the child's second birthday, was found to be strongly associated with improved micronutrient intake, child growth and cognitive development (Lutter et al., 2018; Neumann et al., 2003). Despite these health and nutrition benefits, ASF consumption in LMICs remains low, partly due to their low availability, accessibility and affordability (Baye & Kennedy, 2020).

Despite sustained nutrition education, dietary diversity in Ethiopia has remained low (Baye, 2021). According to the most recent Demographic and Health Survey (DHS), only 12.5% of children 6–23 months of age met the minimum dietary diversity score (Baye et al., 2020). Although poultry production is common in rural households and eggs are the cheapest among ASFs, egg consumption has remained suboptimal as they are still out of reach for a large segment of the population (Hailemichael et al., 2016). A recent synthesis of evidence showed that the primary barrier for consumption of eggs was their high price relative to incomes, making eggs unaffordable for a large majority of Ethiopians (Hirvonen et al., 2020). The high price of eggs relative to incomes is, at least in part, a reflection of the weak market integration, seasonality of demands and the high transaction cost (e.g., breakage of eggs). Addressing these consumption barriers requires increasing egg production but also calls for innovations in the food system.

Innovations that can improve the egg value chain, reduce transaction costs and food losses (e.g., breakage) and increase the shelf‐life of eggs, if supported with effective dietary advises, can increase egg products consumption levels and, in turn, promote the egg value chain. More importantly, increased consumption of eggs leads to improved child growth, improved early cognitive development and can contribute to the satisfaction of energy and nutrient requirements (Lutter et al., 2018). One such innovation is the drying of eggs into egg powder using spray‐drying techniques. Drying eggs into powder can reduce transport and storage costs, increase the shelf life and allow smaller portions to be added as ingredients in habitual Ethiopian diets (Abreha, Getachew, Laillou, Chitekwe, & Baye, 2021). Therefore, if the availability, accessibility and the demand of egg powder are increased, dietary and nutrient adequacy may increase, accordingly. However, this potential has not been systematically evaluated.

This study aimed to evaluate the contribution of integrating egg powder to the nutrient adequacy and the affordability of diets. To this end, we simulated the incorporation of whole egg powder into households and children's diet and evaluated its contribution to the nutrient adequacy and affordability of nutritious diets for households and children aged 6–23 months. We linked the target group dietary requirements, regional food prices and household consumption data and applied linear programming modelling to determine the minimum‐cost diet, its affordability and the potential impact of incorporating egg powder into household's diet.

## 2 METHODS

### 2.1 Data sources

**Figure 1:**
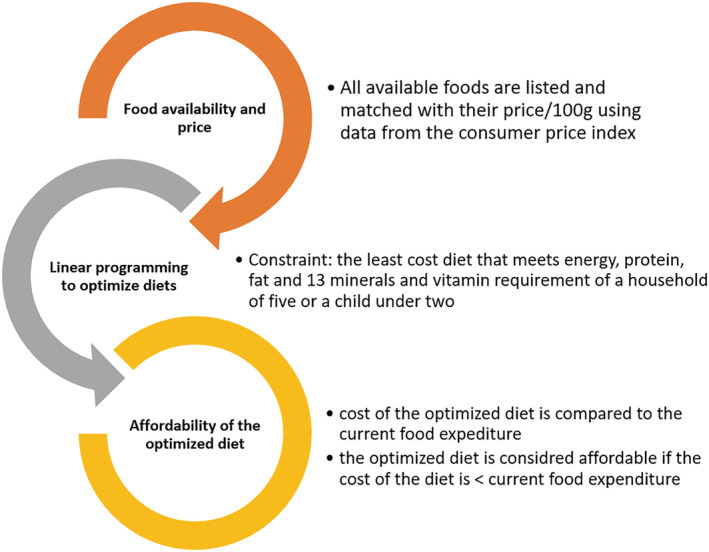
The Fill the Nutrient Gap approach

We used nationally and regionally representative data from nine regions and two administrative cities of Ethiopia to estimate the minimum‐cost nutritious diet and the extent to which whole egg powders can contribute to the affordability of the optimized diet. First, we used the household consumption and expenditure survey (HCES) to estimate the share of income allocated for egg consumption. We then integrated the locally available foods to their price and nutrient composition and used the ‘Fill the Nutrient Gap’ approach using the cost of the diet (CoTD) software. We estimated the minimum‐cost nutritious diet without (control) and with whole egg powder integrating into households' food basket. We finally estimated the share of the households that can afford the optimized diets. We did not apply the entire cycle of the Fill the Nutrient Gap approach (Figure 1) but rather its core analysis of the cost and nonaffordability of nutritious diets. This was so, because EPHI, with technical support from WFP, had already covered the steps of the FNG approach (EPHI and WFP, 2020). Furthermore, we saw strategic value in expanding the specific FNG Ethiopia section on the use of egg powder. The ‘Fill the Nutrient Gap’ approach is briefly presented in Figure 1.

### 2.2 Study population

We estimated the cost of the minimum‐cost nutritious diet including whole egg powder for (i) household of five members and (ii) a child under 2 years of age. We then compared the estimated minimum‐cost diet with the optimized diet (control) from the Fill the Nutrient Gap project report (EPHI & WFP, 2020). The nutrient requirement for a household of five members was estimated assuming a household composed of (i) one child under 2 years of age, (ii) one school age child, (iii) one adolescent girl, (iv) a pregnant/lactating woman and (v) an adult man. The household composition considered is the same one agreed in the Fill the Nutrient Gap project report (EPHI & WFP, 2020).

### 2.3 Egg consumption by wealth quintile

Using the 2015–2016 HCES data, we calculated the percentage share of the total expenditure allocated for egg consumption. We also estimated the share of the total expenditure that would be required if a household is to consume an egg a day or every other day. The mean share of expenditure was calculated and further disaggregated by wealth quintile.

### 2.4 Price and nutrient composition of whole egg powder

The nutritional value of whole egg powder was taken from a study that characterized spray‐dried eggs from local and exotic breeds in Ethiopia (Abreha et al., 2021). For nutrients that were not characterized, values from the USDA nutrient database were borrowed (https://fdc.nal.usda.gov/). The price of whole egg powder was calculated for a processing capacity of 15,000 eggs/day/plant, taking into account the cost of raw materials, utilities, packaging, human resources, transport and profit margins (Table S1). Variations in price of raw eggs, profit margins and transport cost were considered. This led to an estimated price of 6.0–6.8 Ethiopian Birr (ETB) per egg equivalent of whole egg powder (~10 g). These price estimates were calculated from real data sourced from two processors planning to process whole egg powder in Ethiopia. The average nutrient composition of 2.5‐g whole egg powder is presented in Table S2.

### 2.5 Estimation of the optimal portion size of whole egg powder

We determined the portion size that would allow a minimum‐cost diet that fulfils the energy and nutrient requirements. A whole egg powder portion size constraint of 0–7.5 (min–max) g per day was set, but the linear programming determined 2.5 g/person/day as the optimal portion that can allow the minimum‐cost nutritious diet to be achieved (Table 1). A 2.5‐g portion costed about 1.5–1.7 ETB and could allow a child 6–23 months of age to fulfil 62.5% of the choline and 47% of the protein requirements from complementary foods (Lutter & Dewey, 2003).

**Table 1:** Composition of the optimized diet by food group

	Food group	Minimum‐cost nutritious diet g/day/person (%)	
		Without egg powder	With egg powder
1	Grains and grain‐based products	403.3 (44)	416.3 (53)
2	Roots and tubers	7.2 (1)	12.3 (2)
3	Legumes, nuts and seeds	39.9 (4)	41.3 (5)
4	Meat	0.0 (0)	0.0 (0)
5	Fish and sea foods	9.2 (1)	9.2 (1)
6	Eggs and egg products^a^	40.1 (4)	2.5 (0.3)
7	Milk and milk products	88.9 (10)	115.6 (14.8)
8	Vegetables and vegetable products	310.3 (34)	164.2 (21)
9	Fruits and fruits products	0.0 (0)	0.0 (0)
10	Oils and fats	14.1 (2)	17.9 (2)
11	Sugars and confectionary	0.0 (0)	0.0 (0)
12	Herbs, spices and condiments	0.0 (0)	0.0 (0)

^a^ The linear programming entirely replaced ‘eggs and egg products’ by egg powder as a cheaper alternative in the optimized diet option including egg powder.

### 2.6 Minimum‐cost nutritious diet with and without whole egg powder

The minimum‐cost nutritious diet was estimated based on a list of locally available foods (for every region), assuming average portion sizes and price. Price of foods was collected by the Central Statistics Agency, collected from 119 consumer price index (CPI) markets, which were grouped by regions and city administrations. More detailed descriptions of these estimations can be found elsewhere (EPHI & WFP, 2020). To be considered nutritious, the diet needed to meet energy, protein, fat and 13 micronutrients including vitamins and minerals. The nutrients included were calcium, iron, magnesium, zinc, niacin, pantothenic acid, vitamin A, C, B1, B2, B6, B9 and B12. The minimum possible cost of the nutritious diet was calculated using linear programming using the CoTD software (Deptford et al., 2017). The minimum‐cost nutritious diet was run for two scenarios: (1) based on locally available foods (without whole egg powder) and (2) with the integration of 2.5‐g whole egg powder/person/day into the food basket of the household. The price difference between the above two scenarios was expressed in percentage.

### 2.7 Affordability of the minimum‐cost nutritious diet

The minimum monthly cost of the nutritious diet without‐ (control) and with whole egg powder (intervention) was calculated and then compared with the total food expenditure of the household. A diet was defined as affordable if the CoTD was less than or equal to the current food expenditure. We accounted for inflation between the two surveys: the CPI (2018/2019) and the HCES (2015–2016).

To calculate affordability of the minimum‐cost diet for a child under 2 years of age, we first calculated the total food expenditure in any household and then in households with a child under 2 years of age, separately. Subsequently, we attributed the difference between the two estimates as the food expenditure allocated for the child. We then compared this food expenditure with the cost of the minimum‐nutritious diet. The average share (%) of the minimum‐cost diet relative to the food expenditure allocated for the child was estimated, and its distribution by wealth quintile was calculated. The effect of a free distribution of whole egg powder on the affordability of the minimum‐cost nutritious diet was also evaluated.

## 3 RESULTS

**Figure 2:**
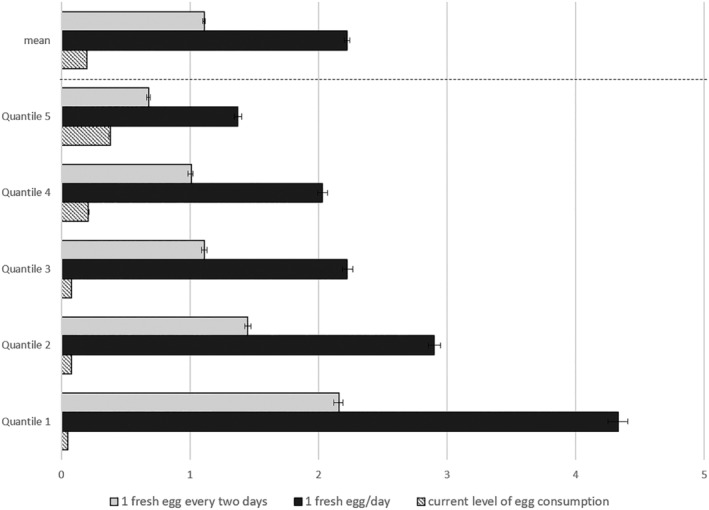
Share of household expenditure (%) spent and needed for egg consumption. Error bars represent 95% confidence intervals. *Source*: Authors' analyses of HCES, 2015–2016

The share of households' total consumption expenditure required to allow a household to consume an egg a day or every other day is presented in Figure 2. Consumption of a fresh egg a day requires the average household to spend up to 2.2% of the total consumption expenditure, whereas every other day, consumption requires 1.1% of the consumption expenditure. These figures are 5–10 times higher than the current level of expenditure for eggs (0.2%). Disaggregating the figures by wealth quintile reveals that the poorest wealth quintile needs to spend up to 4.3% and 2.6% of its total household expenditure to consume an egg per day or every other day, respectively. For the poorest quintile, the current level of expenditure (0.05%) should be multiplied by 52 and 92 to allow consumption of an egg a day or every other day, respectively. In contrast, the richest wealth quintile needs to multiply the current share of expenditure allocated for egg (0.38%) by 1.8 and 3.6, to allow consumption every other day and every day, respectively.

**Figure 3:**
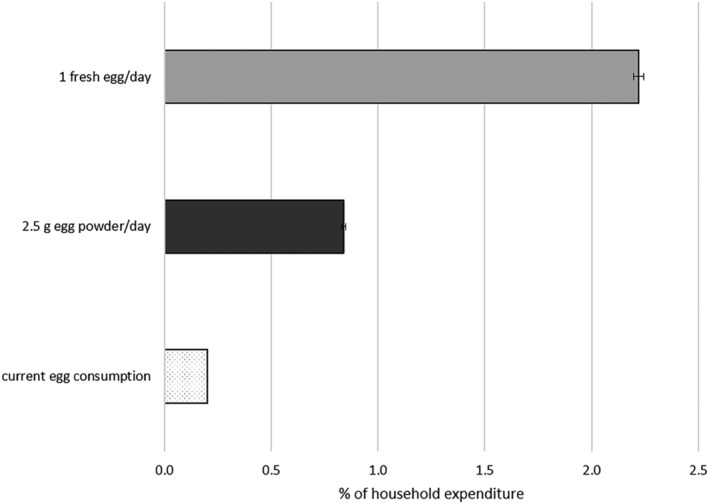
Share of household expenditure (%) needed for different portions of egg and egg powder. Error bars represent 95% confidence intervals. *Source*: Authors' analyses of HCES, 2015–2016

Assuming that whole egg powders in sachets of 2.5 g are made widely available, the everyday consumption of whole egg powder would require the average households to allocate a much higher proportion (0.84%) than their current expenditure (0.20%), but this is a much lower figure than what would be required to consume a fresh egg everyday (2.2%) or every other day (1.1%; Figure 3).

**Figure 4:**
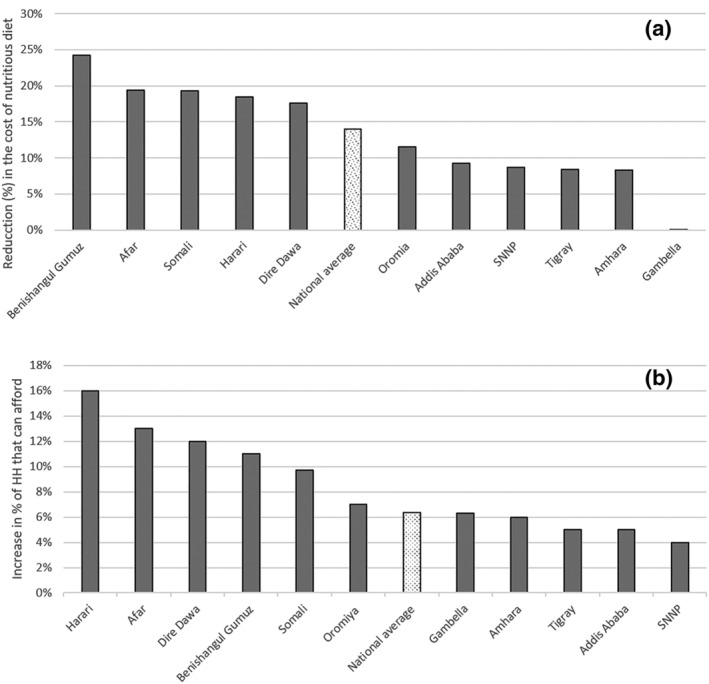
Reduction in the cost (a) and increase in proportion of household that can afford (b) the minimum‐cost nutritious diet with the integration of 2.5 g of egg powder/day. *Source*: Authors' Fill the Nutrient Gap analysis

Noteworthy is the reduction in the cost of the minimum‐cost nutritious diet when whole egg powders become an option at 1.7birr for 2.5 g of powder (Figure 4a,b). The cost of the optimized minimum‐cost nutritious diet (baseline) can be reduced by 14% at the national level, when 2.5 g of whole egg powder was added to the list of food options. The effect of the introduction of whole egg powders is highly variable by region, with cost reductions exceeding 15% for regions like Benishangul Gumuz, Afar, Somali and Harari. The cost reductions in Tigray and Amhara regions (which account for the highest stunting prevalence in Ethiopia) were estimated to be 8%, whereas Gambela region will not see any reductions in the minimum‐cost nutritious diet. However, such reductions in the minimum‐cost nutritious diet will allow, at the national level, an additional 6% households (~4–6 million individuals) to afford the minimum‐cost nutritious diet when whole egg powder is made available and accessible than in the baseline scenario (without egg powder).

**Figure 5:**
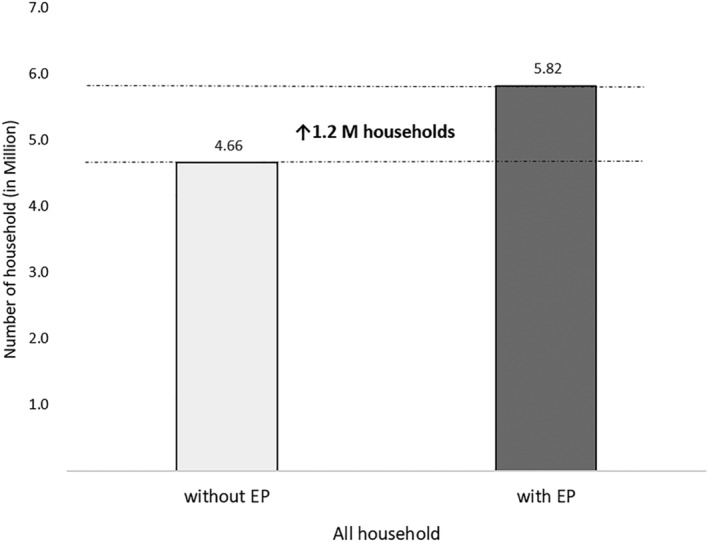
Number of households (in millions) that can afford the minimum‐cost nutritious diet before and after integration of egg powder (EP) into the market

The proportion of households that benefits from the reduction of the minimum‐cost nutritious diet varies from 4% in SNNP to 16% in Harari. This is an additional ~1.2 million households affording the minimum‐cost nutritious diet (Figure 5), representing 4–6 million individuals depending on the size of the households. However, even the reduced minimum‐cost diet requires a substantial share of the household expenditure, when considering the most vulnerable five‐member household that includes nutritionally vulnerable pregnant/lactating women, children under 2 and adolescents.

Focusing on households with children less than 2 years of age and calculating the share of food expenditure allocated for one child show that except for the poorest wealth quintile, the minimum‐cost nutritious diet (baseline and including egg powder) can be affordable (<100% of the total food expenditure; Table 2). Household in the poorest wealth quintile can only afford the minimum‐cost nutritious diet, if whole egg powder is to be provided for free.

**Table 2:** Share (%) of food expenditure needed to be spent for a child (6–23 months) to consume the minimum nutritious cost diet by household wealth quintile

Wealth quintile	Total food expenditure (ETB)		Food expenditure (for U2 child)				
	Mean (95% CI)			% of food expenditure for HH with U2 child			
	All HH	HH with U2		CoTD baseline	CoTD_2.5 g EP 1.71ETB	CoTD_2.5 g EP 1.5 ETB	CoTD_2.5 g EP free
Poorest	14,053 (13,777, 14,328)	15,487 (14,939, 16,036)	1,434 (1162, 1708)	134.6 (113.0, 166.2)	128.9 (108.2, 159.0)	127.4 (106.9, 157.2)	87.9 (73.8, 108.4)
Second	19,400 (19,039, 19,761)	21,573 (20,852, 22,294)	2,173 (1813, 2,533)	88.9 (76.2, 106.5)	85.0(73.0, 101.9)	84.0 (72.1, 100.7)	58.0 (49.7, 69.5)
Middle	25,313 (24,819, 25,806)	28,489 (27,490, 29,489)	3,176 (2,671, 3,683)	60.8 (52.4, 72.3)	58.2 (50.2, 69.2)	57.5 (49.6, 68.4)	39.7 (34.2, 47.2)
Fourth	28,031 (27,431, 28,630)	33,389 (31,921, 34,857)	5,358 (4490, 6227)	36.0 (31.0, 43.0)	34.5 (29.7,41.2)	34.1 (29.3,40.7)	23.5 (20.2,28.1)
Richest	34,063 (33,336, 34,790)	45,816 (43,856, 47,777)	11,753 (10,520, 12,987)	16.4 (14.9, 18.4)	15.7 (14.2, 17.6)	15.5 (14.1, 17.4)	10.7 (9.7, 12.0)
National	24,171 (23,912, 24,430)	26,293 (25,721, 26,864)	21,229 (1809, 2434)	91.0 (79.3, 106.7)	87.1 (75.9, 102.2)	86.1 (75.0, 101.0)	59.4 (51.8, 69.7)

Abbreviations: CoTD, minimum cost of the nutritious diet; EP, egg powder; ETB, Ethiopian Birr; HH, households; U2, child under 2 years of age.

## 4 DISCUSSION

In view of the low egg consumption in Ethiopia, this study appointed to an innovative solution that can increase the levels of egg consumption. Fresh egg consumption requires a significant share of the total diet‐related expenditure, particularly in the poorest households (last quintile). We evaluated the impact of including whole egg powder into a five‐member household's diet on the cost and affordability of the least‐cost, nutrient adequate diet. Our model suggests that whole egg powder can lower the cost of the minimum‐cost nutritious diet by an average of 16 percentage points, implying that whole egg powder can provide potential programmatic strategies to increase egg consumption and improve the overall dietary quality of children aged 6–23 months.

The consumption of nutrient‐dense foods like ASF has been consistently associated with lower risk of micronutrient deficiencies, improved child linear growth and cognitive development (Headey et al., 2018; Lutter et al., 2018). This realization led to nutrition messaging that consistently advises the consumption of a diverse diet that includes at least one ASF (Gebremedhin et al., 2017; World Health Organization [WHO], 2003). Nevertheless, even the cheapest form of ASF, eggs, have not been regularly consumed, neither by infants and young children that need it the most. Looking at the share of the expenditure needed to consume one egg a day and comparing it with current levels of expenditure, it is clear that egg consumption even on every other day basis is beyond the reach of many households. In line with this observation, the minimum‐cost nutritious diet is unaffordable by 76% of the Ethiopian households, which is comparable with the most recent State of Food and nutrition Insecurity (SOFI 2020) report that shed light on the stark reality that a great proportion of the world population cannot afford a nutrient‐adequate diet (Herforth et al., 2020).

If the aspiration of ending malnutrition is still to be upheld, increasing incomes and making nutritious foods accessible and affordable are critical. With less than a decade away from the sustainable development goals (SDGs) and the long‐term consequence that malnutrition can have, a food systems' transformation that make nutritious and healthy diets accessible/affordable is direly needed. Dehydrating eggs can provide such opportunities by increasing the nutrient density of diets, increasing shelf‐life, reducing losses, creating job and income opportunities, to name a few (Abreha et al., 2021). Our simulation of including whole egg powder into the food basket of households' diets has allowed us to further push downward the cost of the already optimized minimum‐cost nutritious diet, allowing an additional 6% households (~4–6 million individuals) afford the diet. However, this was still not enough to benefit a large share of households in Ethiopia, reminding us the need to also improve the affordability of other nutrient‐dense foods like fruits and vegetables (Bachewe et al., 2017).

Although income levels are still not enough to afford the reduced cost nutritious diet that includes whole egg powder, focusing on children less than 2 years of age rather than the entire household suggests otherwise. Assuming that the additional food expenditure that households with under 2 children spent was entirely on the child, the expenditure levels were enough to afford the minimum‐cost nutritious diet, except for the poorest wealth quintile. However, the poorest wealth quintile would need free distribution through a cash transfer value (in the shape of cash or voucher system), leveraging the existing social protection programme. Noteworthy is that our simulation suggests that such support, if supplemented with effective demand generation for a nutrient‐dense diet, can allow even children from the poorest wealth quintile to afford the minimum‐cost nutritious diet.

The whole egg powder helps concentrate nutrients in a small volume and thus responds to a critical need for young children that have small gastric capacity and high energy/nutrient needs to support their growth (WHO, 2003). The whole egg powder can also allow households to provide eggs to their children during the Orthodox fasting periods without fear of ‘contaminating’ utensils (Haileselassie et al., 2020). Although the processing of egg into powder requires additional costs, the divisibility of egg powder into smaller portions, the reduction in transport cost, the increased shelf‐life and the prevention of breakage can make egg powder more cost‐effective. Hence, our price estimates, not considering all these anticipated benefits, suggest that the price of egg powder could be further lowered. Besides, a backward integration of egg processing to production has the potential to increase the productivity of the egg sector, leading to further reductions in egg price (Beesabathuni et al., 2018; Hirvonen et al., 2020).

The present study has a number of limitations that need to be considered when interpreting our findings. First, although a long list of macro‐ and micronutrients were considered in defining the minimum‐cost nutritious diet, it is not comprehensive enough to consider all nutrients of interests (e.g., essential amino acids). Second, demand‐related aspect was not considered, although its importance should not be underestimated since egg powder would represent a ‘new’ food to the traditional diet in Ethiopia. The estimates of the nonaffordability of a nutritious diet relied on the latest available household expenditure data (2016), but this may not reflect changes in expenditure in recent years.

Notwithstanding the above limitations, our study suggests that the simple dehydration of egg into egg powder has potential for a substantial contribution towards increasing egg consumption, reducing the cost of nutritious diet and increasing its affordability, if supported by a robust demand generation strategy. These findings can inform the design of more effective nutrition and social protection programmes that aim to make nutritious diets more accessible and affordable.

## CONFLICT OF INTEREST

The authors declare that they have no conflicts of interest.

## CONTRIBUTIONS

KB, AA and AL conceived the study with inputs from SC and FD; KB, AA and PG prepared and analysed the data; KB and AA wrote the paper with inputs from AL, SC, PG and FD. All authors read and approved the final manuscript.

## Supporting information


**Figure S1.** Flow diagram of data required by the Cost of the Diet Software to optimize the cost of meeting energy and nutrients requirement of individuals or groups
**Source:** adopted from Deptford et al., 2017
**Table S1** Example of a summary of a price calculation of whole egg powder
Various scenario including raw material cost (3–5 ETB/egg) variation, profit margin (10–15%) and transport cost, were considered.
**Table S2** Average energy and nutrient composition (/100 g) of egg‐powder.
Source: Abreha et al., (2021) complemented with additional data from USDA nutrient database: https://fdc.nal.usda.gov/
**Figure S2** The fill the nutrient gap conceptual framework
Source: Bose et al, 2019

## Data Availability

The household consumption and economic survey can be accessed from the Ethiopian Central Statistics Agency, upon request.

## References

[mcn13274-bib-0001] Abreha, E. , Getachew, P. , Laillou, A. , Chitekwe, S. , & Baye, K. (2021). Physico‐chemical and functionality of air and spray dried egg powder: Implications to improving diets. International Journal of Food Properties, 24(1), 152–162. 10.1080/10942912.2020.1867569

[mcn13274-bib-0002] Bachewe, F. , Hirvonen, K. , Minten, B. , & Yimer, F. (2017). The Rising Costs of Nutritious Foods in Ethiopia (p. 67). IFPRI ESSP Research Note.

[mcn13274-bib-0003] Baye, K. (2021). Improved diet quality, a missing ingredient for accelerating stunting reduction: An example from Ethiopia. Archives of Disease in Childhood, archdischild‐2020‐320292. 10.1136/archdischild-2020-320292 33402327

[mcn13274-bib-0004] Baye, K. , & Kennedy, G. (2020). Estimates of dietary quality in infants and young children (6–23 mo): Evidence from demographic and health surveys of 49 low‐and middle‐income countries. Nutrition, 78, 110875. 10.1016/j.nut.2020.110875 32653760

[mcn13274-bib-0005] Baye, K. , Laillou, A. , & Chitweke, S. (2020). Socio‐economic inequalities in child stunting reduction in sub‐Saharan Africa. Nutrients, 12(1), 253. 10.3390/nu12010253 31963768 PMC7019538

[mcn13274-bib-0006] Beesabathuni, K. , Lingala, S. , & Kraemer, K. (2018). Increasing egg availability through smallholder business models in East Africa and India. Maternal & Child Nutrition, 14, e12667. 10.1111/mcn.12667 30332537 PMC6865889

[mcn13274-bib-0007] Deptford, A. , Allieri, T. , Childs, R. , Damu, C. , Ferguson, E. , Hilton, J. , Parham, P. , Perry, A. , Rees, A. , Seddon, J. , & Hall, A. (2017). Cost of the Diet: A method and software to calculate the lowest cost of meeting recommended intakes of energy and nutrients from local foods. BMC Nutrition, 3(1), 1–17. http://www.fao.org/, 10.1186/s40795-017-0136-4 PMC7050783 32153808

[mcn13274-bib-0008] EPHI , WFP . (2020). Fill the Nutrient Gap Ethiopia Validation of preliminary results. (December 2020), 0–29.

[mcn13274-bib-0009] Gebremedhin, S. , Baye, K. , Bekele, T. , Tharaney, M. , Asrat, Y. , Abebe, Y. , & Reta, N. (2017). Predictors of dietary diversity in children ages 6 to 23 mo in largely food‐insecure area of South Wollo, Ethiopia. Nutrition, 33, 163–168. 10.1016/j.nut.2016.06.002 27499206

[mcn13274-bib-0010] Hailemichael, A. , Gebremedhin, B. , Gizaw, S. , & Tegegne, A. (2016). Analysis of village poultry value chain in Ethiopia: Implications for action research and development. LIVES Working Paper 10, ILRI.

[mcn13274-bib-0011] Haileselassie, M. , Redae, G. , Berhe, G. , Henry, C. J. , Nickerson, M. T. , Tyler, B. , & Mulugeta, A. (2020). Why are animal source foods rarely consumed by 6‐23 months old children in rural communities of Northern Ethiopia? A qualitative study. PLoS ONE, 15(1), e0225707. 10.1371/journal.pone.0225707 31914130 PMC6948827

[mcn13274-bib-0012] Headey, D. , Hirvonen, K. , & Hoddinott, J. (2018). Animal sourced foods and child stunting. American Journal of Agricultural Economics, 100(5), 1302–1319. 10.1093/ajae/aay053 33343003 PMC7734193

[mcn13274-bib-0013] Herforth, A. , Bai, Y. , Venkat, A. , Mahrt, K. , Ebel, A. , & Masters, W. (2020). Cost and affordability of healthy diets across and within countries: Background paper for The State of Food Security and Nutrition in the World 2020 (Vol. 9). FAO Agricultural Development Economics Technical Study No. 9. Food & Agriculture Org.

[mcn13274-bib-0014] Hirvonen, K. , Baye, K. , Headey, D. D. , & Hoddinott, J. F. (2020). Value Chains for Nutritious Food: Analysis of the Egg Value Chain in the Tigray Region of Ethiopia (Vol. 152). Intl Food Policy Res Inst.

[mcn13274-bib-0015] Krasevec, J. , An, X. , Kumapley, R. , Bégin, F. , & Frongillo, E. A. (2017). Diet quality and risk of stunting among infants and young children in low‐and middle‐income countries. Maternal & Child Nutrition, 13, e12430. 10.1111/mcn.12430 29032628 PMC6865990

[mcn13274-bib-0016] Lutter, C. K. , & Dewey, K. G. (2003). Proposed nutrient composition for fortified complementary foods. The Journal of Nutrition, 133(9), 3011S–3020S. 10.1093/jn/133.9.3011S 12949402

[mcn13274-bib-0017] Lutter, C. K. , Iannotti, L. L. , & Stewart, C. P. (2018). The potential of a simple egg to improve maternal and child nutrition. Maternal & Child Nutrition, 14, e12678. 10.1111/mcn.12678 30332538 PMC6865885

[mcn13274-bib-0018] Moursi, M. M. , Arimond, M. , Dewey, K. G. , Treche, S. , Ruel, M. T. , & Delpeuch, F. (2008). Dietary diversity is a good predictor of the micronutrient density of the diet of 6‐to 23‐month‐old children in Madagascar. The Journal of Nutrition, 138(12), 2448–2453. 10.3945/jn.108.093971 19022971

[mcn13274-bib-0019] Neumann, C. G. , Bwibo, N. O. , Murphy, S. P. , Sigman, M. , Whaley, S. , Allen, L. H. , Guthrie, D. , Weiss, R. E. , & Demment, M. W. (2003). Animal source foods improve dietary quality, micronutrient status, growth and cognitive function in Kenyan school children: Background, study design and baseline findings. The Journal of Nutrition, 133(11), 3941S–3949S. 10.1093/jn/133.11.3941S 14672294

[mcn13274-bib-0020] World Health Organization . (2003). Complementary feeding: Report of the global consultation, and summary of guiding principles for complementary feeding of the breastfed child.

