# Supplementary material for: Whole egg powder makes nutritious diet more affordable for Ethiopia: A cost of the diet and affordability analysis
Source: Matern Child Nutr. 2021 Sep 23;20(Suppl 5):e13274. doi: 10.1111/mcn.13274 (PMC11258759; doi:10.1111/mcn.13274)
Supplement: Supplementary file 1 — Figure S1. Flow diagram of data required by the Cost of the Diet Software to optimize the cost of meeting energy and nutrients requirement of individuals or groups Source: adopted from Deptford et al., 2017 Table S1 Example of a summary of a price calculation of whole egg powder Various scenario including raw material cost (3–5 ETB/egg) variation, profit margin (10–15%) and transport cost, were considered. Table S2 Average energy and nutrient composition (/100 g) of egg‐powder. Source: Abreha et al., (2021) complemented with additional data from USDA nutrient database: https://fdc.nal.usda.gov/ Figure S2 The fill the nutrient gap conceptual framework Source: Bose et al, 2019 [file MCN-20-e13274-s001.docx]

**Supplement**

**+**

**Inclusion of egg-powder**

Affordability

Income and expenditure data

Cost of the diet

Foods to meet nutrient requirements

Recommended intake of protein and micronutrients

Portion sizes

Min and max % of energy from fat

Dietary habits

Average energy requirement

Cost per 100 g

Individuals or families

Local foods

Intake of nutrients and energy

Food tables

Requirements

Nutrients

## **Figure S1.** Flow diagram of data required by the Cost of the Diet Software to optimize the cost of meeting energy and nutrients requirement of individuals or groups

## **Source:** adopted from Deptford et al. 2017

| **Cost estimation for 15,000 eggs processed** | cost/egg  (10g powder) | 2.5 g egg powder |
| --- | --- | --- |
| Raw materials | 4.00 | 1.0 |
| Utilities | 0.01 | 0.0 |
| Labor | 0.21 | 0.1 |
| Total | 4.21 | 1.1 |
| Packaging (20%) | 0.84 | 0.2 |
| Profit margin +transport cost (10%) | 0.42 | 0.1 |
| **TOTAL** | **5.48** | **1.4** |

**Table S1** Example of a summary of a price calculation of whole egg powder

Various scenario including raw material cost (3-5 ETB/egg) variation, profit margin (10-15%) and transport cost, were considered.

**Table S2** Average energy and nutrient composition (/100 g) of egg-powder.

|  | **Nutrient composition (/100 g)** | |
| --- | --- | --- |
| **Proximate** | min | max |
| Moisture (g/100g) | 2.6 | 3.4 |
| energy (kcal/100g) | 565 | 580 |
| Protein (g/100g) | 44.3 | 45.9 |
| Fat (g/100g) | 36.3 | 39.1 |
| Utilizable carbohydrate (g/100g) | 12.6 | 13.6 |
| **Macro-minerals (mg)** |  | |
| Calcium | 326 | 341 |
| Magnesium | 82 | 92 |
| Potassium | 818 | 834 |
| Sodium | 457 | 488 |
| Phosphorus | 143 | 181 |
| **Trace-minerals (mg)** |  | |
| Iron | 11 | 14 |
| Zinc | 1.2 | 2.6 |
| Copper | 0.26 | 0.27 |
| Manganese | 0.12 | 0.16 |
| iodine, ug | 0 |  |
| selenium, ug | 123.2 |  |
| **Vitamins** |  |  |
| vitamin A, ug RE | 640 |  |
| biotin, ug | 0 |  |
| choline, mg | 1176 |  |
| folic acid, ug | 188 |  |
| niacin, mg | 0.304 |  |
| panthotenic acid, mg | 6.128 |  |
| riboflavin, mg | 1.824 |  |
| thiamin, mg | 0.16 |  |
| vitamin B6, mg | 0.68 |  |
| vitamin B12, ug | 3.56 |  |
| vitamin C, mg | 0 |  |
| vitamin D, ug | 8 |  |
| vitamin E, mg | 4.2 |  |

Source: Abreha et al., (2021) complemented with additional data from USDA nutrient database: <https://fdc.nal.usda.gov/>


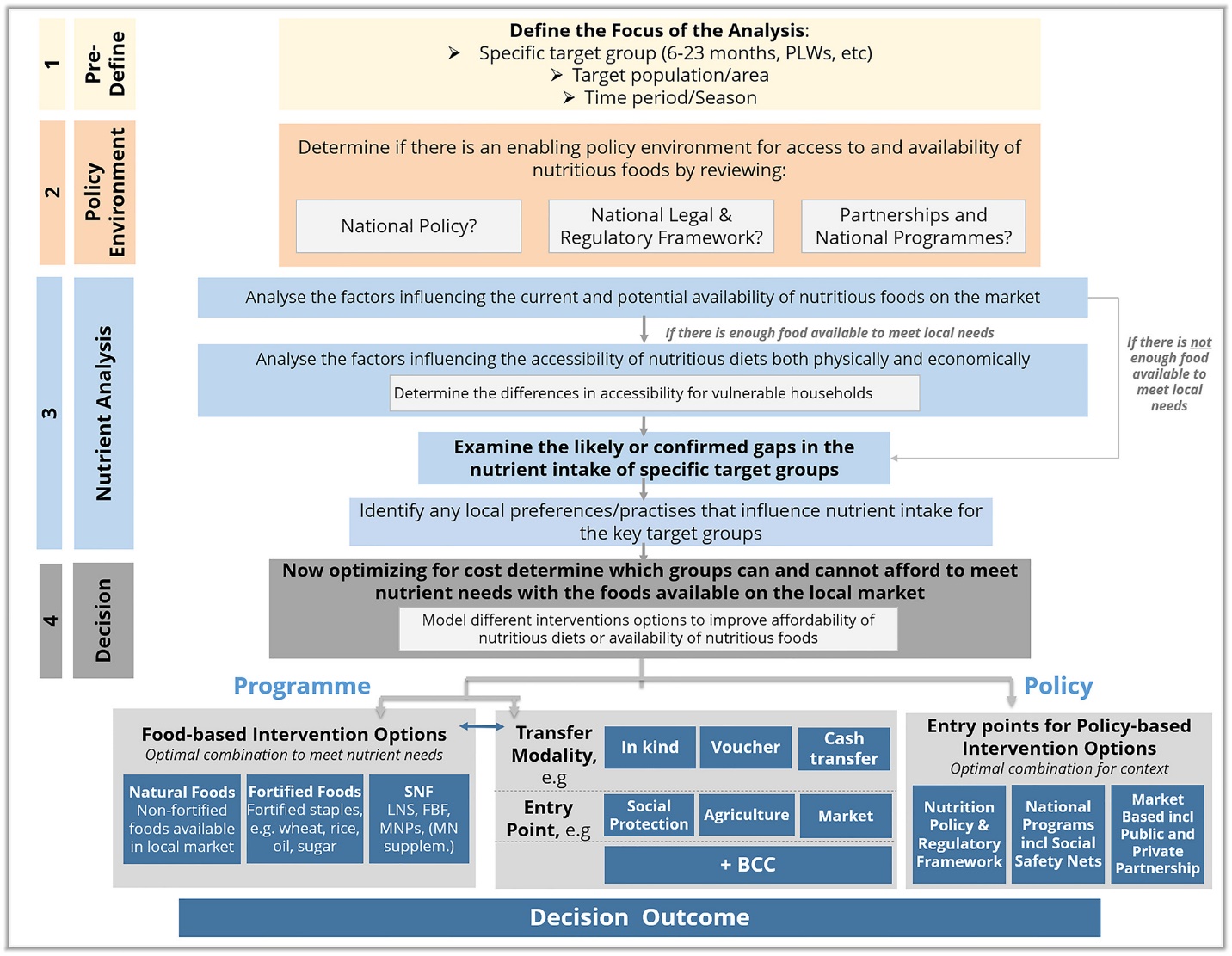


**Figure S2** The fill the nutrient gap conceptual framework

Source : Bose et al, 2019
